# Oleic Acid and Linoleic Acid Enhances the Biocontrol Potential of *Metarhizium rileyi*

**DOI:** 10.3390/jof10080521

**Published:** 2024-07-26

**Authors:** Guang Wang, Xu Zhang, Guangzu Du, Wenqian Wang, Yunhao Yao, Sitong Jin, Haosheng Cai, Yuejin Peng, Bin Chen

**Affiliations:** Yunnan State Key Laboratory of Conservation and Utilization of Biological Resources, College of Plant Protection, Yunnan Agricultural University, Kunming 650201, China; wang_gunag_17@126.com (G.W.); zhxv08@163.com (X.Z.); duguangzu1986@163.com (G.D.); wqwangts@163.com (W.W.); yaoyunh@163.com (Y.Y.); jinsitong1015@163.com (S.J.); cai_hao_sheng@163.com (H.C.)

**Keywords:** growth, development, stress tolerances, virulence

## Abstract

*Metarhizium rileyi* is a wide spread insect fungi with a good biocontrol potentiality to various pests, particularly noctuid insects. However, it is characterized by its slow growth, its sensitivity to abiotic stress, and the slow speed of kill to pests, which hinder its use compared with other entomopathogenic fungi. In this study, the responses of *M. rileyi* to eight types of lipids were observed; among the lipids, oleic acid and linoleic acid significantly promoted the growth and development of *M. rileyi* and enhanced its stress tolerances and virulence. An additional mechanistic study demonstrated that exogenous oleic acid and linoleic acid significantly improved the conidial germination, appressorium formation, cuticle degradation, and cuticle infection, which appear to be largely dependent on the up-regulation of gene expression in growth, development, protective, and cuticle-degrading enzymes. In conclusion, exogenous oleic acid and linoleic acid enhanced the stress tolerances and virulence of *M. rileyi* via protecting conidial germination and promoting cuticle infection. These results provide new insights for the biopesticide development of *M. rileyi*.

## 1. Introduction

*Metarhizium* (Nomuraea) *rileyi* (Farlow) Kepler (Hypocreales: Clavicipitaceae) is an entomopathogenic fungus that shows a strong virulence against Nymphalidae, Noctuidae, and other pests [1,2,3,4,5], such as *Spodoptera frugiperda*, *Spodoptera litura*, *Spodoptera cosmioides*, and *Chrysodeixis includens*. *M. rileyi* can control crop pests in different agricultural systems, as shown by greenhouse and field trials [6,7,8]. However, *M. rileyi* is slow growing, sensitive to abiotic stress, and slow to kill pests, hindering its use compared with other entomopathogenic fungi (*Beauveria bassiana* and *Metarhizium anisopliae*) and decreasing its viability, biological activity, and prevalence under field conditions [8,9,10,11]. Therefore, enhancing the resistance of *M. rileyi* to abiotic stresses and improving its speed of killing pests are critical for its industrial-scale use as a bioinsecticide.

Conidia are the main infection and reproductive units of entomopathogenic fungi [12]. Maintaining or improving the viability, biological activity, and abundance of conidia under field conditions is the main strategy for applying entomopathogenic fungi. Many methods of improving the performance of conidia have been reported, such as regulating the culture substrate [13], adding exogenous oil or surfactants [14,15], and adapting to abiotic stress [16]. Oil suspensions are one of the most common types of pesticide formulations. Oil suspension formulations of conidia made from vegetable oil or mineral oil can significantly improve the pathogenicity and abiotic stress tolerance of the conidia [14,15]. Conidial oil suspensions of *M. rileyi* can efficiently control *S. frugiperda* in corn fields [17]. Furthermore, vegetable oil can improve the conidial germination, yield, and thermotolerance of *B. bassiana*, *Simplicillium lanosoniveum*, and *Isaria fumosorosea* [18,19,20]. Compared to mineral oil, vegetable oil has the function of regulating the growth and development of entomopathogenic fungi [14,15,18,19,20], suggesting that there is a certain substance in vegetable oil that is beneficial for conidial growth and development.

The main components of vegetable oil are lipids and esters generated from glycerol [21], including palmitic acid (C16:0, 4.6–20.0%), oleic acid (C18:1, 6.2–71.1%), and linoleic acid (C18:2, 1.6–79.0%), which are present in coconut oil, castor oil, olive oil, sunflower oil, and palm oil [21]. In fungal species such as *Cordyceps*, *Metarhizium*, *Beauveria*, and others, lipids can serve as a protective agent for conidia against stresses and signaling molecules for growth and development [10,19,22,23]. Linoleic acid and oleic acid increased the conidial tolerance to heat, drought, and oxidative stresses [10,19,24], which promoted spore germination, the branching of hyphae, pseudohyphal growth, conidial production, and the transcription of growth- and development-related genes [22,23]. Notably, lipids are one of the main components of oxylipins; they change the phospholipid composition, membrane homeostasis, membrane fluidity, and conidial hydrophobicity (an important indicator of the initial cuticle infection) [25,26,27]. These changes are involved in improving germination, conidiogenesis effects, fungal proliferation, pathogenicity, and stress tolerance [25,26,27,28]. Thus, we speculate that lipids may have important functions in the growth, development, virulence, and stress tolerance of entomopathogenic fungi. Despite many previous studies investigating the effect of lipids on the abiotic stress tolerance and conidial yield of *M. rileyi*, the mechanism of lipids in improving conidial quality remains poorly understood.

Therefore, the objectives of this study were as follows: (1) to investigate the effects of eight lipids on the growth and development of *M. rileyi*; (2) to determine the role of oleic acid and linoleic acid in the stress tolerance and pathogenicity of *M. rileyi*; and (3) to evaluate the mechanism through which oleic acid and linoleic acid improve the stress tolerance, growth and development, and pathogenicity of *M. rileyi*.

## 2. Materials and Methods

### 2.1. Fungal Strain and Media

*M. rileyi* strain SZCY201010 was isolated from infected *S. frugiperda* larvae in a corn field in Shizong county, Qujing city, Yunnan province, China, and was cultured on Sabouraud maltose agar plus yeast extract medium (SMAY: 1% peptone, 1% yeast extract, 4% maltose, and 1.5% agarose; Coolaber, Beijing, China), as previously described [29]. The media plates were incubated at 25 °C with a photoperiod consisting of a 12L:12D cycle in a culture chamber. Conidial suspensions were prepared from fungi grown on SMAY plates for 10 days.

### 2.2. Phenotype Assays

Fungal growth and development: Aliquots of 5 μL of a conidial suspension (1 × 10^5^, 1 × 10^6^, and 1 × 10^7^ conidia/mL) were inoculated onto SMAY with 0.1% lipids, including oleic acid, linoleic acid, stearic acid, eicosane, cetane, palmitic acid, tetracosane, and octacosanol (Aladdin, Shanghai, China; chemical formula provided in Figure S1). The plates were incubated at 25 °C in a 12L:12D cycle within a culture chamber at 25 °C. Daily observations were made of each colony’s yeast-like blastospore formation time (YG), hyphal growth time (HG), and sporulation time (ST). The diameters of all the colonies incubated for 5, 7, 9, and 11 days at 25 °C were measured. The experiment was performed in triplicate, with three parallel controls each time. The relative growth rate (GR) was estimated with the formula (d_t2_ − d_t1_)/(t2 − t1) × 100 (dt_1_, starting colony diameter; dt_2_, end colony diameter).

Fungal stress responses: Aliquots of 5 μL of a conidial suspension (1 × 10^7^ conidia/mL) were inoculated onto SMAY alone (CK) or supplemented with H_2_O_2_ (3 mM) for oxidative stress; NaCl (0.5 M; Coolaber, Beijing, China), KCl (0.5 M; Coolaber, Beijing, China), glycerol (1 M; Coolaber, Beijing, China), and sorbitol (0.5 M; Coolaber, Beijing, China) for osmotic stress; and calcofluor white (5 μg/mL; Coolaber, Beijing, China) for cell wall stress. The diameters of all the colonies incubated for 10 days under the stresses at 25 °C were measured as described above. The colony response to heat shock was examined by exposing normal 2-day-old SMAY colonies to 42 °C for 1–3 h. The colonies were transferred to 25 °C for 8-day growth recovery, followed by measuring the colony diameters. This experiment was performed in triplicate, with three parallel controls each time. The percentage of relative growth inhibition (RGI) was estimated with the formula (d_c_ − d_t_)/d_c_ × 100 (d_c_, control colony diameter; d_t_, stressed colony diameter) and used as an index of hyphal sensitivity to each stress [11].

Conidial germination: Conidial suspensions (1 × 10^7^ conidia/mL, 100 μL) were spread onto SMAY alone (control) or supplemented with 0.190% oleic acid, 0.175% linoleic acid, or different types of stresses. All the treatments were incubated at 25 °C and the germination percentage was determined for 100 conidia. The entire experiment was performed in triplicate.

Yeast-like blastospores: Aliquots of 5 μL of a conidial suspension (1 × 10^7^ conidia/mL) were inoculated onto SMAY alone (control) or supplemented with 0.190% oleic acid or 0.175% linoleic acid. The plates were cultured at 25 °C with a photoperiod of a 12L:12D cycle in a culture chamber. The yeast-like blastospore yield of *M. rileyi* on the SMAY plates was measured at 3, 4, and 5 days. The entire experiment was performed five times.

Conidia production: To evaluate the conidial yield of *M. rileyi*, *M. rileyi* conidia were suspended in a 0.02% Tween 80 solution (1 × 10^6^ and 1 × 10^7^ conidia/mL). Aliquots of a 100 μL conidial suspension were spread onto SMAY plates with varying concentrations (0.01, 0.05, 0.1, 0.2, and 0.4%) of oleic acid and linoleic acid. The plates were cultured at 25 °C with a photoperiod of a 12L:12D cycle in a culture chamber. When the color of the colony changed from pale or olive green to malachite green, the conidial yield of *M. rileyi* on the SMAY plates was evaluated at 10 d. Three replicates were used for each treatment.

### 2.3. Fungal Virulence-Related Phenotypes

Simulation of host cuticle conidial germination: To view conidial germination on water agar plates (1.5% agarose), conidial suspensions (1 × 10^7^ conidia/mL, 100 μL) were spread onto the plates. Disinfected and sterilized *Locusta migratoria manilensis* hindwings and water agar plates prepared in high-temperature and -pressure facilities were used to mimic the host cuticle and oligotrophic conditions, respectively. A conidial suspension (1 × 10^7^ conidia/mL) was sprayed onto the locust hindwings. All the treatments were incubated at 25 °C and the germination percentage was determined for 100 conidia. Three replicates were used for each treatment.

Appressorium induction: Appressorium formation assays were carried out according to previous reports [30]. Conidia were harvested in 0.01% (vol/vol) Triton X-100 from 10 d old fungal cultures on SMAY. A 20 μL conidial suspension (5 × 10^8^ conidia/mL) was added to 4 mL of MM-Gly (6 g/L NaNO_3_, 0.52 g/L KCl, 0.52 g/L MgSO_4_·7H_2_O, 0.25 g/L KH_2_PO_4_, and 10 mL glycerol; Coolaber, Beijing, China) in plastic plates (diameter = 6 cm) and incubated at 27 °C for 48 or 72 h for appressorium induction. We selected 100 conidia to calculate the appressorium formation rate and performed three biological replicates.

Cuticle-degrading enzyme: Extracellular enzyme activity assays were performed according to the method described in previous reports by Gebremariam et al. [31], and appropriate modifications were made. The conidia were made into a 1 × 10^7^ conidia/mL suspension, alone or supplemented with 0.190% (*v*/*v*) oleic acid or 0.175% (*v*/*v*) linoleic acid. Aliquots of 5 μL of the conidial suspension were inoculated onto 1/8 SMAY supplemented with 1.2% powdered skim milk (*v*/*v*; Yili, Huhehaote, China), 0.2% olive oil (*v*/*v*; Sangon Biotech, Shanghai, China), and 0.5% colloidal chitin (*w*/*w*; coolaber, Beijing, China) to measure the protease, chitinase, or lipase activity, respectively. The plates were incubated at 25 °C for 14 days. The determination of enzyme activity was based on the transparent hydrolysis zone around the colony. The diameter of the colonies and hydrolysis zones was measured using the cross method. The enzymatic index (EI) was calculated using the following formula: EI = (hydrolysis zone diameter)/(colony growth diameter). The entire experiment was performed five times.

### 2.4. Bioassays for Fungal Virulence

The *S. frugiperda* larvae that were used for virulence determination were 3rd, 4th, and 5th instars. A Tween 80 solution (0.02%) was used alone as the control (CK) or supplemented with 0.190% (*v*/*v*) oleic acid (OA) or 0.175% (*v*/*v*) linoleic acid (LA). The conidia were made into a 1 × 10^8^ conidia/mL suspension comprising *M. rileyi* conidia alone (Mr) or supplemented with 0.190% (*v*/*v*) oleic acid (Mr+OA) or 0.175% (*v*/*v*) linoleic acid (Mr+LA). Healthy 3rd-, 4th-, and 5th-instar larvae of the same size were selected, put into the prepared conidial suspension for 15 s, and then removed; the excess water on the surface of the insect body was removed with aseptic filter paper. Subsequently, the larvae were placed individually into a disinfected and sterilized 12-well cell culture plate with 75% alcohol. A fresh artificial diet (125 g of soybean flour, 225 g of corn flour, 225 g of corn leaf flour, 40 g of yeast extract, 20 g of casein, 0.6 g of cholesterol, 3 g of choline chloride, 6 g of sorbic acid, 6 g of methyl p-hydroxybenzoate, 36 g of agarose, 0.1 g of inositol, 7 g of vitamin C, and 1300 mL of water) was added to the holes where the larvae were placed. The above experiment was performed three times, with three treatments for each experiment and 30–36 larvae for each treatment. After the treatment, the larvae were fed the artificial diet and maintained in an artificial climate chamber at 25 °C with a relative humidity of 75% and a photocycle of 16L:8D. The number of dead insects was recorded each day, and the dead insects were kept in a Petri dish to determine whether they were infected with the test strain.

The blastospore growth was observed to examine the time it took to penetrate the cuticle. The hemolymph of five 4th-instar larvae in the Mr, Mr+OA, and Mr+LA infection groups was collected and diluted 1:1 in a sterile physiological solution (0.14 M NaCl, 0.1 M glucose, 25 mM sodium citrate, and 30 mM citric acid). The hyphal bodies were photographed and observed under a microscope every 12 h from 48 to 132 h post-inoculation of the conidia. The average of five fields was taken as one biological replicate. Five replicates were used for each treatment.

### 2.5. CAT, POD, and SOD Activity Assays

*M. rileyi* was cultured separately in the SMY culture media (1% peptone, 1% yeast extract, and 4% maltose), alone or supplemented with 0.190% (*v*/*v*) oleic acid or 0.175% (*v*/*v*) linoleic acid. These liquid culture media were inoculated with a final concentration of 10^7^ conidia/mL and cultured for 6 days at 25 °C on a rotary with a speed of 200 rpm. The mycelium was filtered and washed four times with PBS. A BCA protein content assay kit, catalase (CAT) activity assay kit, peroxidase (POD) activity assay kit, and superoxide dismutase (SOD) activity assay kit (Boxbio Science & Technology Co., Ltd., Beijing, China) were used to determine the protein concentration and total enzymatic activity (U/mg protein). Three replicates were used for each treatment.

### 2.6. Quantitative Real-Time Reverse-Transcription Polymerase Chain Reaction (RT-qPCR)

Aliquots of 100 μL of the conidial suspension (1 × 10^7^ conidia/mL) were smeared onto SMAY alone (CK) or supplemented with 0.190% oleic acid or 0.175% linoleic acid and incubated at 25 °C for 5 days for the determination of gene expression. The total RNA was isolated using the TRIzol reagent (BBI Life Sciences, Shanghai, China) by following the manufacturer’s instructions. The first-strand cDNA was synthesized from the total RNA using a HiScript III All-in-One RT SuperMix Perfect for qPCR (Vazyme, Nanjing, China). The RT-qPCR analysis was carried out in 96-well 0.2 mL block plates using a QuantStudio™ 7 Flex system (Thermo Scientific, Wilmington, DE, USA). Each reaction contained 1.0 μL of the template (1 ng/μL), 10.0 μL of 2 × Taq Pro Universal SYBR qPCR Master Mix (Vazyme, Nanjing, China), and 0.4 μL of each primer (10 μM), and 8.2 μL of EDPC-ddH_2_O, with a final volume of 20 μL. The RT-qPCR condition was pre-denaturation at 95 °C for 30 s, and then 40 cycles at 95 °C for 5 s and at 60 °C for 20 s. After each reaction, a melting curve analysis (denatured at 95 °C for 15 s, annealed at 60 °C for 60 s, and denatured at 95 °C for 15 s) was conducted to ensure the consistency and specificity of the amplified product. Three biological replicates were used for each treatment in the RT-qPCR analyses. The β-tubulin gene (TUB) and transcription elongation factor (TEF) genes were employed as internal references [32,33]. The 2^−ΔΔCT^ technique was utilized to determine the relative expression of every gene [34]. Table S1 contains a list of primers utilized in the RT-qPCR tests.

### 2.7. Statistical Analysis

All the data are presented as the mean ± standard deviation. The data were analyzed for the normality of their distribution using the Shapiro–Wilk test and for homoscedasticity using Levene’s test. When these assumptions were not fulfilled, the data were subjected to a log or square root transformation. If the data met the assumptions of normality and homogeneity, two groups were analyzed using an unpaired Student’s *t*-test and data among the various groups were analyzed using a one-way analysis of variance (ANOVA) followed by Tukey’s post hoc test. If the data were not normal and/or not homogenous, two groups were analyzed using a Wilcox test and the data among the various groups were analyzed using the non-parametric Kruskal–Wallis test, followed by the Games–Howell test. The survival data were subjected to a Kaplan–Meier survival log-rank analysis. The median lethal time (LT_50_) for certain treatments was calculated using linear regression in Microsoft Excel, version 2019. *p* < 0.05 was considered to indicate statistical significance.

## 3. Results

### 3.1. Effects of Lipids Addition on M. rileyi Growth and Development

**Figure 1:**
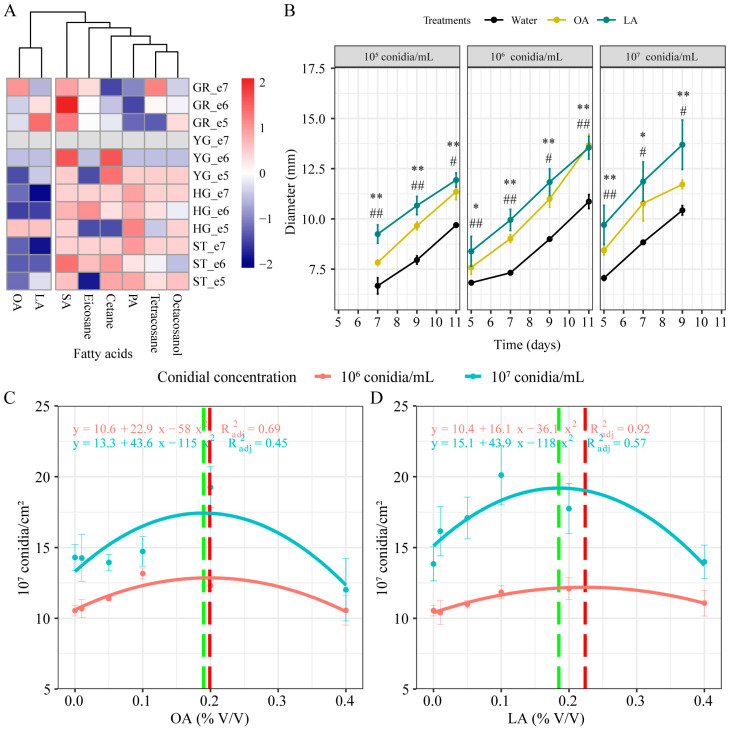
Effects of lipids addition on *M. rileyi* growth and development. (**A**) Cluster heat map and clustering through the level of growth and development. Red and blue boxes indicate increased and decreased levels, respectively. LA, linoleic acid; OA, oleic acid; SA, stearic acid; PA, palmitic acid; GR, relative growth rate; YG, yeast cell formation time; HG, hyphal growth time; ST, sporulation time; e5, 10^5^ conidia/mL; e6, 10^6^ conidia/mL; e7, 10^7^ conidia/mL. (**B**) Colony diameter. The conidial production of *M. rileyi* at different concentrations of oleic acid (**C**) and linoleic acid (**D**) was determined and curve fitting (y = c + bx + ax^2^) was used to determine the optimal concentration. The conidial yield for the 10^6^ conidia/mL suspension is shown by the red line and the conidial yield for the 10^7^ conidia/mL suspension is shown by the green line. GT_50_, conidial median germination time. The data are presented as the mean ± standard deviation. SDs of the means are from three independent replicates. * *p* < 0.05 and ** *p* < 0.01 when the OA group is compared to the water group. ^#^ *p* < 0.05 and ^##^ *p* < 0.01 when the LA group is compared to the water group.

The growth and development rhythm of *M. rileyi*, including conidial germination, yeast-like blastospore production, mycelial growth, and sporulation, was determined (Figure S1). We used a heat map clustering analysis based on the yeast-like blastospore formation time, hyphal growth time, sporulation time, and relative growth rate data to compare the effects of different lipids. As shown in Figure 1A, oleic acid and linoleic acid clustered into one branch, while other lipids (SA, eicosane, cetane, PA, tetracosane, and octacosanol) clustered into another. Exogenous oleic acid and linoleic acid significantly increased the colony diameter in the conidial suspensions with concentrations of 10^5^, 10^6^, and 10^7^ conidia/mL (Figure 1B; Table S2). Next, we measured the conidial production of *M. rileyi* at different concentrations of oleic acid and linoleic acid and used curve fitting (y = c + bx + ax^2^) to determine the optimal concentration, where y is the conidial production and x is the concentration. The optimal concentration of oleic acid was 0.199% and 0.190% for concentrations of 10^6^ and 10^7^ conidia/mL, respectively (Figure 1C; Table S3). The optimal concentration of linoleic acid was 0.224% and 0.175% for concentrations of 10^6^ and 10^7^ conidia/mL, respectively (Figure 1D; Table S3). Thus, the concentrations of 0.190% oleic acid and 0.175% linoleic acid were selected for further study.

### 3.2. Exogenous Oleic Acid and Linoleic Acid Enhanced the Growth and Development of M. rileyi

**Figure 2:**
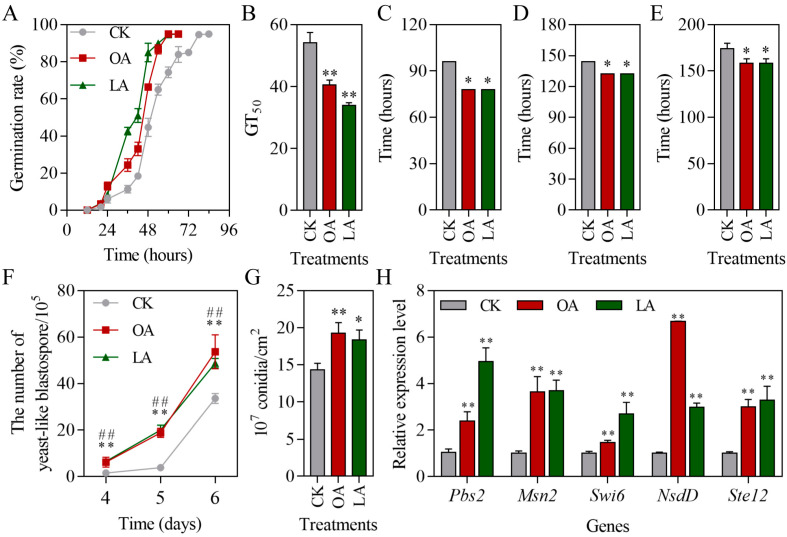
Exogenous oleic acid and linoleic acid enhanced the growth and development of *M. rileyi*. (**A**) Conidial germination curve. (**B**) Conidial median germination time. (**C**) Yeast-like blastospore formation time. (**D**) Hyphal growth time. (**E**) Sporulation time. (**F**) The number of yeast-like blastospores after 4, 5, and 6 days. (**G**) The conidial yield at 10 days. (**H**) The expression of growth- and development-related genes. LA, linoleic acid; OA, oleic acid; Pbs2, mitogen-activated protein kinase kinase Pbs2; Msn2, zinc finger DNA-binding protein Msn2; Swi6, cell cycle box-binding transcription factor MrSwi6; NsdD, GATA-type transcription factor NsdD; Ste12, transcription factor Ste12; TEF, transcription elongation factor; TUB, β-tubulin. The data are presented as the mean ± standard deviation. A-E, G, and H: SDs of the means from three independent replicates. F: SDs of the means from five independent replicates. * *p* < 0.05 and ** *p* < 0.01 when the OA group is compared to the control (CK) group; ^##^ *p* < 0.01 when the LA group is compared to the control (CK).

The growth and the development of *M. rileyi* on SMAY alone or SMAY with 0.190% oleic acid or 0.175% linoleic acid were observed. Exogenous oleic acid and linoleic acid significantly enhanced the conidial germination compared with the CK group (Figure 2A). The GT_50_ of the CK group was 54.2 ± 3.3 h, which was significantly higher than that of the exogenous oleic acid group (40.5 ± 1.6 h; Student’s *t*-test, *p* = 0.0030) and linoleic acid group (34.0 ± 0.8 h; Student’s *t*-test, *p* = 0.0005; Figure 2B). In addition, these exogenous fatty acids (OA and LA) significantly reduced the yeast-like blastospore formation time (Figure 2C; Wilcox test, *p* = 0.0254), hyphal growth time (Figure 2D; Wilcox test, *p* = 0.0254), and sporulation time (Figure 2E; Student’s *t*-test, *p* = 0.0245). The number of yeast-like blastospores in all the groups increased over time, and it was significantly increased in the exogenous oleic acid group after 4 (Wilcox test, *p* = 0.0088), 5 (Wilcox test, *p* = 0.0088), and 6 (Wilcox test, *p* = 0.0090) days of incubation (Figure 2F). Exogenous linoleic acid also significantly increased the number of yeast-like blastospores at 4 (Student’s *t*-test, *p* < 0.0000), 5 (Wilcox test, *p* = 0.0090), and 6 (Student’s *t*-test, *p* < 0.0000) days of incubation (Figure 2F). After 10 days of cultivation, exogenous oleic acid (Student’s *t*-test, *p* = 0.0074) and linoleic acid (Student’s *t*-test, *p* = 0.0130) notably raised the conidia production compared with the CK group (Figure 2G). Furthermore, we determined the expression of growth- and development-related genes (*MrPbs2*, *MrMsn2*, *MrSwi6*, *MrNsdD*, and *MrSte12*) [23]. Exogenous oleic acid significantly up-regulated the expression of *MrPbs2* (Student’s *t*-test, *p* = 0.0055), *MrMsn2* (Student’s *t*-test, *p* = 0.0024), *MrSwi6* (Student’s *t*-test, *p* = 0.0041), *MrNsdD* (Student’s *t*-test, *p* < 0.0000), and *MrSte12* (Student’s *t*-test, *p* = 0.0005; Figure 2H). Similarly, the expression of *MrPbs2* (Student’s *t*-test, *p* = 0.0004), *MrMsn2* (Student’s *t*-test, *p* = 0.0006), *MrSwi6* (Student’s *t*-test, *p* = 0.0047), *MrNsdD* (Student’s *t*-test, *p* < 0.0000), and *MrSte12* (Student’s *t*-test, *p* = 0.0028) was significantly increased in the linoleic acid supplementation group (Figure 2H).

### 3.3. Exogenous Oleic Acid and Linoleic Acid Enhanced the Multiple Stress Tolerances of M. rileyi

**Figure 3:**
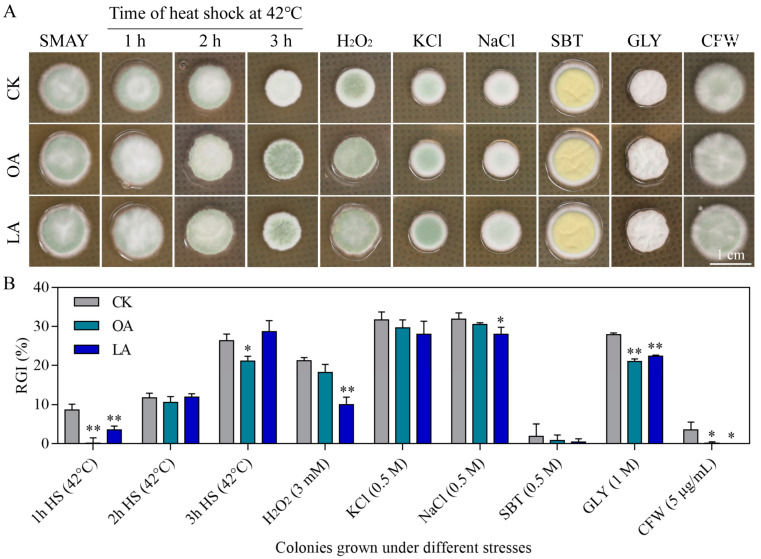
Exogenous oleic acid and linoleic acid enhanced multiple stress tolerances of *M. rileyi*. Images (**A**) and relative growth inhibition percentages (**B**) of fungal colonies incubated at 25 °C for 10 days on SMAY plates or supplemented with the indicated concentrations of H_2_O_2_, KCl, NaCl, sorbitol (SBT), glycerol (GLY), and calcofluor white (CFW) and of SMAY colonies incubated at 25 °C for 8 days of growth recovery after 2-day-old colonies were exposed to a 42 °C heat shock (HS) for 1, 2, or 3 h. Scale bars = 1.0 cm; LA, linoleic acid; OA, oleic acid; RGI, relative growth inhibition; HS, heat shock. The data are presented as the mean ± standard deviation. SDs of the means are from three independent replicates. * *p* < 0.05 and ** *p* < 0.01 compared to the control (CK) response.

Exogenous oleic acid and linoleic acid resulted in increased tolerances of *M. rileyi* to various stress cues during 10-day colony growth on SMAY plates exposed to chemical stressors and a 42 °C heat shock for 1–3 h during the period of incubation (Figure 3). During the 10 days of incubation (Figure 3A), the *M. rileyi* colonies in all the treatment groups produced olive green conidia on SMAY with 1 h and 2 h of heat shock, H_2_O_2_, KCl, NaCl, and CFW, as well as pale yellow conidia and a white mycelium on SBT and GLY, respectively. After 3 h of heat shock, the *M. rileyi* in the exogenous oleic acid and linoleic acid groups grew a white mycelium, while that in the CK group only grew a white mycelium in the center of the colony. We also evaluated the RGI on the 10th day of incubation (Figure 3B); compared to the CK group after 1 h of HS (8.7 ± 1.2%), 3 h of HS (26.4 ± 1.4%), GLY (27.9 ± 0.4%), and CFW (3.5 ± 1.6%), exogenous oleic acid significantly reduced the RGI in the 1 h (0.1 ± 1.1%; Student’s *t*-test, *p* = 0.0018) and 3 h (21.1 ± 1.1%; Student’s *t*-test, *p* = 0.0125) heat shock, GLY (21.0 ± 0.5%; Student’s *t*-test, *p* = 0.0001), and CFW (0.2 ± 0.3%; Student’s *t*-test, *p* = 0.0474) groups. Compared to the CK group after 1 h of HS (8.7 ± 1.2%), H_2_O_2_ (21.3 ± 0.6%), NaCl (31.9 ± 1.3%), GLY (27.9 ± 0.4%), and CFW (3.5 ± 1.6%), exogenous linoleic acid significantly reduced the RGI in the 1 h HS (3.5 ± 0.8%; Student’s *t*-test, *p* = 0.0071), H_2_O_2_ (10.0 ± 1.6%; Student’s *t*-test, *p* = 0.0007), NaCl (28.0 ± 1.5%; Student’s *t*-test, *p* = 0.0487), GLY (22.4 ± 0.20%; Student’s *t*-test, *p* < 0.0000), and CFW (0.0 ± 0.0%; Student’s *t*-test, *p* = 0.0386) groups.

### 3.4. Exogenous Oleic Acid and Linoleic Acid Protected Conidial Germination of M. rileyi and Improved Stress Tolerance

**Figure 4:**
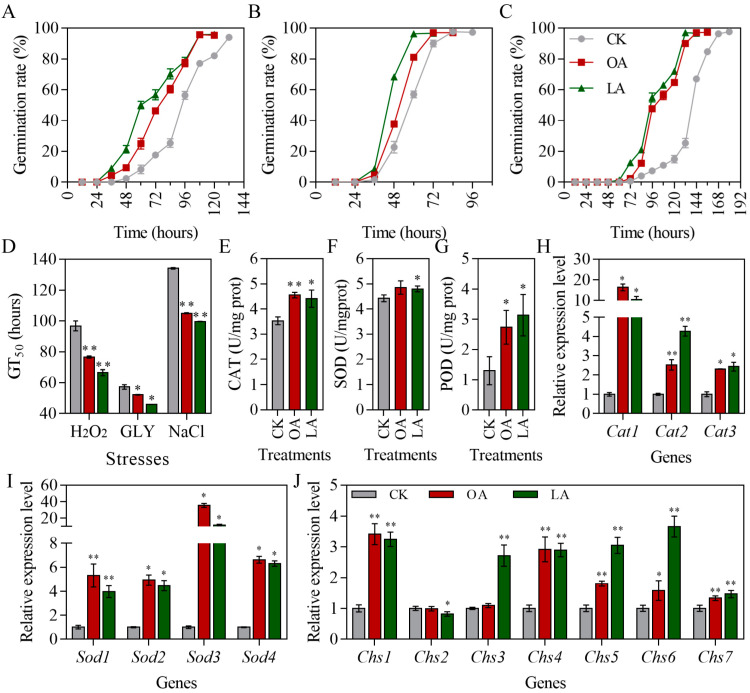
Exogenous oleic acid and linoleic acid protected conidial germination of *M. rileyi* and improved potential for stress tolerance. Conidial germination curve with H_2_O_2_ (**A**), GLY (**B**), and NaCl (**C**) stresses. (**D**) Conidial median germination time. The activity of CAT (**E**), SOD (**F**), and POD (**G**). The expression of genes encoding the CAT (**H**) and SOD (**I**) isoenzymes. (**J**) The expression of genes encoding the chitin synthase isoenzymes. LA, linoleic acid; OA, oleic acid; GT_50_, conidial median germination time; CAT, catalase; SOD, superoxide dismutase; POD, peroxidase; Chs, chitin synthase. The data are presented as the mean ± standard deviation. SDs of the means are from three independent replicates. * *p* < 0.05 and ** *p* < 0.01 when compared to the control (CK).

The germination dynamics of *M. rileyi* conidia under H_2_O_2_, glycerol, and NaCl stresses are shown in Figure 4A, 4B, and 4C, respectively. Exogenous oleic acid and linoleic acid protected the conidial germination of *M. rileyi* under H_2_O_2_, glycerol, and NaCl stresses. The conidial median germination time (GT_50_) under H_2_O_2_ (Student’s *t*-test, *p* = 0.0005), glycerol (Student’s *t*-test, *p* = 0.0037), and NaCl (Student’s *t*-test, *p* < 0.0000) stresses was significantly reduced by exogenous oleic acid compared to that of the CK group (Figure 4D). The linoleic acid groups also significantly reduced the GT_50_ under H_2_O_2_ (Student’s *t*-test, *p* = 0.0002), glycerol (Student’s *t*-test, *p* = 0.0002), and NaCl (Student’s *t*-test, *p* < 0.0000) stresses (Figure 4D). To further explore the potential functions underlying oleic acid and linoleic acid against stresses, we investigated the activity of protective enzymes (CAT, POD, and SOD) in *M. rileyi* induced by exogenous oleic acid and linoleic acid. The activity of CAT (Figure 4E; Student’s *t*-test, *p* = 0.0007) and POD (Figure 4F; Student’s *t*-test, *p* = 0.0265) in *M. rileyi* was increased in the oleic acid group compared to that in the CK group. The activity of CAT (Student’s *t*-test, *p* = 0.0158), POD (Student’s *t*-test, *p* = 0.0182), and SOD (Figure 4G; Student’s *t*-test, *p* = 0.0215) in *M. rileyi* was increased in the linoleic acid group compared to that in the CK group. The expression of *MrCat1* (Wilcox test, *p* = 0.0495), *MrCat2* (Student’s *t*-test, *p* = 0.0006), *MrCat3* (Wilcox test, *p* = 0.0495), *MrSod1* (Student’s *t*-test, *p* = 0.0015), *MrSod2* (Wilcox test, *p* = 0.0495), *MrSod3* (Wilcox test, *p* = 0.0495), and *MrSod4* (Wilcox test, *p* = 0.0495) was significantly increased in the oleic acid group compared to that in the CK group (Figure 4H,I). Similarly to oleic acid group, the expression of *MrCat1* (Wilcox test, *p* = 0.0495), *MrCat2* (Student’s *t*-test, *p* < 0.0000), *MrCat3* (Student’s *t*-test, *p* = 0.0007), *MrSod1* (Student’s *t*-test, *p* = 0.0006), *MrSod2* (Wilcox test, *p* = 0.0495), *MrSod3* (Wilcox test, *p* = 0.0495), and *MrSod4* (Wilcox test, *p* = 0.0495) was significantly increased in the linoleic acid group compared to that in the CK group (Figure 4H,I). Moreover, we measured the expression of the chitin synthase family genes, as they are strongly associated with the stress tolerances of entomopathogenic fungi [35,36]. As shown in Figure 4J, the expression of *MrChs1* (Student’s *t*-test, *p* = 0.0003, *p* = 0.0001), *MrChs4* (Student’s *t*-test, *p* = 0.0013), *MrChs5* (Student’s *t*-test, *p* = 0.0004), *MrChs6* (Student’s *t*-test, *p* = 0.0403), and *MrChs7* (Student’s *t*-test, *p* = 0.0098) was significantly up-regulated in the oleic acid group compared to that in the CK group (Figure 4J). The expression of *MrChs1* (Student’s *t*-test, *p* = 0.0001), *MrChs3* (Student’s *t*-test, *p* = 0.0010), *MrChs4* (Student’s *t*-test, *p* = 0.0002), *MrChs5* (Student’s *t*-test, *p* = 0.0002), *MrChs6* (Student’s *t*-test, *p* = 0.0002), and *MrChs7* (Student’s *t*-test, *p* = 0.0066) was also significantly up-regulated in the linoleic acid group compared to that in the CK group (Figure 4J). The expression of *MrChs2* (Student’s *t*-test, *p* = 0.0361) was down-regulated with linoleic acid supplementation (Figure 4J).

### 3.5. Exogenous Oleic Acid and Linoleic Acid Improved the Virulence of M. rileyi

**Figure 5:**
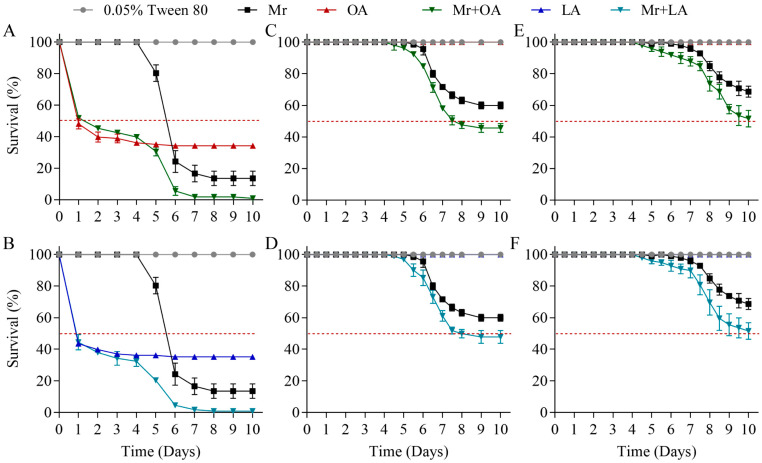
Exogenous oleic acid and linoleic acid improved the virulence of *M. rileyi*. (**A**,**B**) The survival of 3rd instars of *S. frugiperda* exposed to *M. rileyi*. (**C**,**D**) The survival of 4th instars of *S. frugiperda* exposed to *M. rileyi*. (**E**,**F**) The survival of 5th instars of *S. frugiperda* exposed to *M. rileyi*. LA, linoleic acid with a 0.175% concentration; The dashed line represents a survival rate of 50%; OA, oleic acid with a 0.190% concentration; Mr, *M. rileyi* conidial suspension with 10^8^ conidia/mL; Mr+OA, combined treatment of *M. rileyi* and oleic acid; Mr+LA, combined treatment of *M. rileyi* and linoleic acid. The data are presented as the mean ± standard deviation. SDs of the means are from three independent replicates.

The laboratory bioassays revealed a high virulence for the 0.190% oleic acid and 0.175% linoleic acid treatments against the 3rd instars of *S. frugiperda*, with death beginning on the first day and survival reaching 34.3 ± 1.3% and 35.2 ± 1.3% after 6 days (Figure 5A,B; Table S4), respectively. The virulence of *M. rileyi* against the 3rd, 4th, and 5th instars of *S. frugiperda* was determined, with survival reaching 13.6 ± 3.7%, 60.0 ± 1.8%, and 68.7 ± 2.9% (Figure 5; Table S4), respectively, 10 days after the treatment using suspensions of 1 × 10^8^ conidia/mL. The LT_50_ of the 3rd instars treated with *M. rileyi* was 5.7 ± 0.1 d. However, the death rate of the 4th and 5th instars due to *M. rileyi* did not reach 50%, so the LT_50_ could not be calculated. The combined treatment of *M. rileyi* and oleic acid against the 3rd, 4th, and 5th instars of *S. frugiperda* resulted in survival rates of 0.9 ± 1.3%, 45.6 ± 2.3%, and 51.5 ± 4.3%, which were significantly lower than those resulting from the 0.190% oleic acid treatment alone or the *M. rileyi* suspensions alone (Figure 5A,C,E; Table S4). Similarly, the combined treatment of *M. rileyi* and linoleic acid against the 3rd, 4th, and 5th instars of *S. frugiperda* resulted in survival rates of 0.9 ± 1.3%, 47.8 ± 3.4%, and 51.5 ± 4.3%, which were significantly lower than those resulting from the 0.175% linoleic acid treatment alone or the *M. rileyi* suspensions alone (Figure 5B,D,F; Table S4). The LT_50_ for 4th-instar *S. frugiperda* with *M. rileyi*+OA and *M. rileyi*+LA was 7.6 ± 0.2 d and 7.6 ± 0.3 d, respectively.

### 3.6. Exogenous Oleic Acid and Linoleic Acid Increased Fungal Virulence-Related Phenotypes

**Figure 6:**
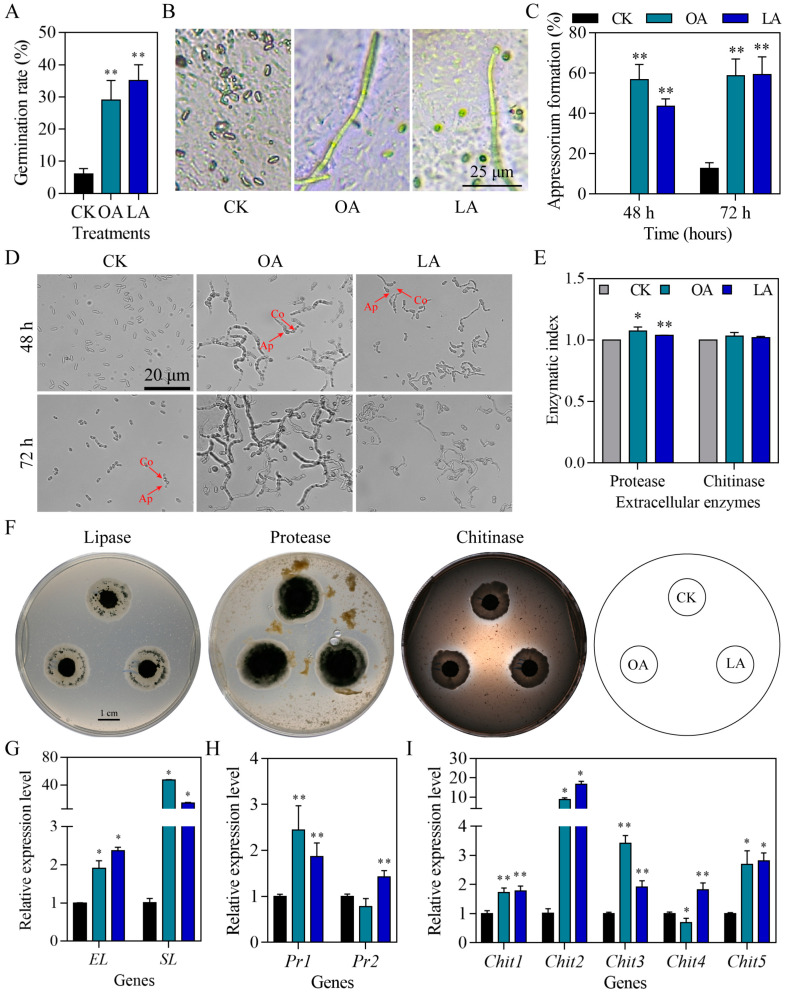
Exogenous oleic acid and linoleic acid increased fungal virulence-related phenotypes. Conidial germination rate on water agar plates (**A**) and locust hindwings (**B**). Appressorium formation rates (**C**) and images (**D**) at 48 and 72 h. The red arrow represents the appressorium. Ap, appressorium; Co, conidia. Extracellular enzymatic index (**E**) and images (**F**) at 14 days. (**G**) The expression of extracellular lipase gene and secreted lipase gene. (**H**) The expression of genes encoding the protease isoenzymes. (**I**) The expression of genes encoding the chitinase isoenzymes. LA, linoleic acid; OA, oleic acid; Chit, chitinase; Pr, protease; EL, extracellular lipase; SL, secreted lipase. The data are presented as the mean ± standard deviation. A, C, and G-I: SDs of the means are from three independent replicates. E: SDs of the means are from five independent replicates. * *p* < 0.05 and ** *p* < 0.01 compared to the control (CK).

The conidial germination on water agar plates was significantly increased in the oleic acid (Student’s *t*-test, *p* = 0.0032) and linoleic acid (Student’s *t*-test, *p* = 0.0007) groups compared with the CK group (Figure 6A). The exogenous oleic acid and linoleic acid groups showed conidial germination on locust hindwings, but the CK group did not show conidial germination (Figure 6B). Exogenous oleic acid significantly improved the appressorium formation rates at 48 (Wilcox test, *p* = 0.0369) and 72 h (Student’s *t*-test, *p* < 0.0000) and accelerated appressorium formation (Figure 6C,D). Linoleic acid also significantly improved the appressorium formation rates at 48 (Wilcox test, *p* = 0.0369) and 72 h (Student’s *t*-test, *p* = 0.0009) and accelerated appressorium formation (Figure 6C,D). Moreover, we detected cuticle-degradation-related extracellular enzyme activity and gene expression. As shown in Figure 6E,F, the exogenous oleic acid (Student’s *t*-test, *p* = 0.0178) and linoleic acid (Student’s *t*-test, *p* < 0.0000) groups significantly increased the extracellular protease activity. However, no differences were identified for extracellular lipase or chitinase. The expression of *MrEL* (extracellular lipase gene; Wilcox test, *p* = 0.0495) and *MrSL* (secreted lipase gene; Wilcox test, *p* = 0.0495) was significantly up-regulated in the oleic acid and linoleic acid groups compared to that in the CK group (Figure 6G). The expression of protease-related genes *MrPr1* (Student’s *t*-test, *p* = 0.0079) and *MrPr2* (Student’s *t*-test, *p* = 0.0081) was significantly increased after linoleic acid supplementation (Figure 6H). Exogenous oleic acid only significantly up-regulated *MrPr1* expression (Student’s *t*-test, *p* = 0.0090). The expression of *MrChit1* (Student’s *t*-test, *p* = 0.0030), *MrChit2* (Wilcox test, *p* = 0.0495), *MrChit3* (Student’s *t*-test, *p* < 0.0000), and *MrChit5* (Wilcox test, *p* = 0.0495) was significantly up-regulated in the oleic acid group compared to that in the CK group, and the expression of *MrChit4* was significantly decreased (Student’s *t*-test, *p* = 0.0308; Figure 6I). Similarly, linoleic acid also significantly up-regulated the expression of *MrChit1* (Student’s *t*-test, *p* = 0.0022), *MrChit2* (Wilcox test, *p* = 0.0495), *MrChit3* (Student’s *t*-test, *p* = 0.0020), *MrChit4* (Student’s *t*-test, *p* = 0.0043), and *MrChit5* (Wilcox test, *p* = 0.0495) compared to the CK group (Figure 6I).

### 3.7. Exogenous Oleic Acid and Linoleic Acid Enhanced Cuticle Infection of M. rileyi

**Figure 7:**
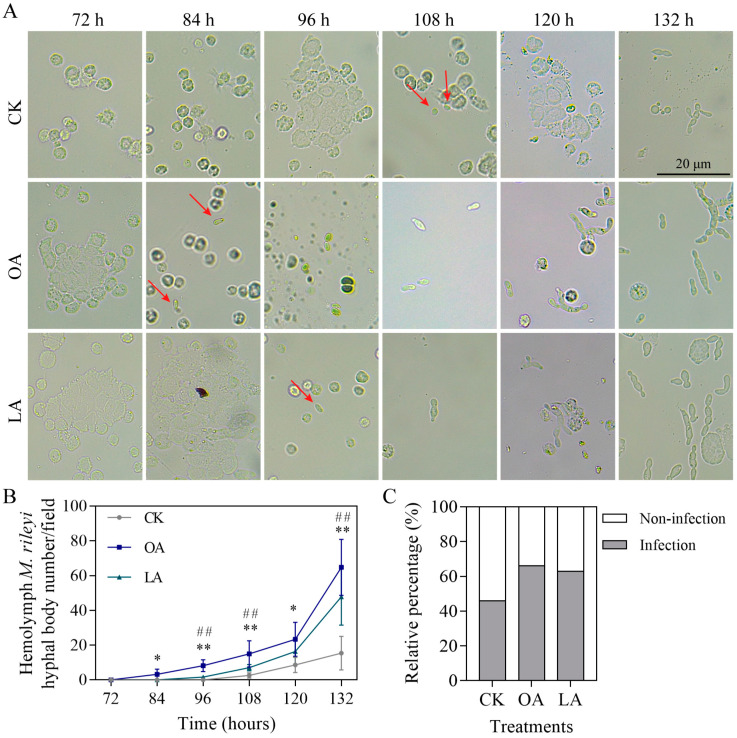
Exogenous oleic acid and linoleic acid enhanced cuticle infection of *M. rileyi*. (**A**) Hemocyte encapsulation was examined under a microscope at an interval of 12 h. Scale bars = 20 μm. The red arrow represents the fungal blastospore in the hemolymph. (**B**) The number of *M. rileyi* hyphal bodies in the hemolymph from 72 to 132 h. (**C**) The relative percentage of infection at 132 h. LA, linoleic acid; OA, oleic acid. The data are presented as the mean ± standard deviation. SDs of the means are from five independent replicates. * *p* < 0.05 and ** *p* < 0.01 when OA is compared to the control (CK); ^##^ *p* < 0.01 when LA is compared to the control (CK).

Exogenous oleic acid and linoleic acid improved the virulence-related phenotypes and increased the cuticle-degradation-related enzyme activity and gene expression in *M. rileyi*, suggesting that exogenous oleic acid and linoleic acid enhance host cuticle infection by *M. rileyi*. After fungal invasion, the host hemocytes flocked together and formed melanic dots. Then, the time for host melanic dots to be created and blastospores to be generated during *M. rileyi* infection was observed. The times of melanic dot formation were 96, 72, and 72 h in the CK, oleic acid, and linoleic acid groups, respectively. The CK, oleic acid, and linoleic acid treatments started to generate blastospores from the host hemocoel at 108, 84, and 96 h, respectively (Figure 7A). The number of blastospores gradually increased from 72 to 132 h, and significantly increased in the oleic acid and linoleic acid treatment groups at multiple time points compared to the CK group (Figure 7B; Table S5). Finally, we observed 30 larvae in each treatment group at 132 h. The infection rates in the CK, oleic acid, and linoleic acid groups were 47, 67, and 63%, respectively (Figure 7C).

## 4. Discussion

Fatty acids have previously been identified as protectors of *M. rileyi* conidia against abiotic stresses, such as dehydration and thermotolerance [10,23,37]. In this study, exogenous oleic acid and linoleic acid protected the *M. rileyi* conidial germination and improved various abiotic stress tolerances, including the tolerance to oxidative, osmotic, cell wall, and heat shock stresses. However, fatty acids can also inhibit the spore germination, hyphal growth, and pathogenicity of entomopathogenic fungi (e.g., *B. bassiana* and *Conidiobolus coronatus*) [38,39,40]. The mechanism for this involves a lipid-droplet protein (*BbLar1*) that maintains the intracellular homeostasis of fatty acids and is crucial to the fungal tolerance to linoleic acid stress, which significantly contributes to fungal compatibility with fatty acids [41]. We found that exogenous oleic acid and linoleic acid significantly increased the activity of protective enzymes and the expression of genes encoding the CAT and SOD isoenzymes. The cumulative evidence proves that the protective enzymes of fungi are crucial to resisting biological and abiotic stress processes [36,42,43], implying that exogenous oleic acid and linoleic acid protect the conidial germination of *M. rileyi* and improve its stress tolerance by increasing the expression of protective enzymes. The cell wall integrity pathway senses hypotonic conditions or multi-stresses, including heat shock, oxidative, osmotic, and cell wall stresses, by controlling cell wall biosynthetic enzymes and the expression of cell-wall-related genes [36,44]. In this study, the transcription level of chitin synthesis genes was significantly up-regulated by the oleic acid and linoleic acid treatments, suggesting that oleic acid and linoleic acid may be signal molecules of the cell wall integrity pathway. These results indicate that fatty acids may function as protector agents of biological and abiotic stresses for *M. rileyi*.

Oil suspension formulations of conidia significantly improve the pathogenicity of entomopathogenic fungi [14,15], among which fatty acids may play an important role [26]. In this study, exogenous oleic acid and linoleic acid significantly improved the virulence of *M. rileyi* against the 4th and 5th instars of *S. frugiperda*. Exogenous oleic acid and linoleic acid significantly elevated the conidial germination on water agar plates and locust hindwings and the appressorium formation rates. After adhering to the cuticle of insects, a fungal conidium germinates and differentiates into a specialized infection structure called an appressorium [12,30,45]. Appressorium-mediated cuticle penetration is accompanied by the combined action of cuticle-degrading enzymes (protease, lipase, and chitinase) [31,46]. Our results also showed that the protease activity was significantly elevated after the oleic acid and linoleic acid treatments. Similarly, protease genes were significantly up-regulated in the oleic acid and linoleic acid groups. No differences were found for extracellular lipase or chitinase. However, the expression of the extracellular lipase gene, secreted lipase gene, and chitinase genes was significantly up-regulated in the oleic acid and linoleic acid groups. This may have been due to the insufficient sensitivity of the plate enzyme activity detection method. Furthermore, vegetable oil has the function of regulating the growth and development of entomopathogenic fungi [14,15,18,19,20]. Triacylglycerides is one of the main components of oil suspension, and is hydrolyzed into fatty acids and glycerol by lipases [47]. In addition, exogenous fatty acids also can enter cells through fatty acid-binding protein. Degradation of fatty acids by the β-oxidation pathway results in the formation of acetyl-CoA, which enters the tricarboxylic acid (TCA) cycle for the production of ATP. TCA cycle intermediates may be used as key sources of carbon molecules for biosynthesis of nucleotides, amino acids, and lipids [48]. Notably, the oleic acid and linoleic acid treatments improved the conidial germination rate on water agar plates and locust hindwings and increased the growth and development of *M. rileyi* [23], suggesting that exogenous oleic acid and linoleic acid provide energy substance for *M. rileyi*’s growth and development [23,30]. Combined with the results of this study, we speculate that oleic acid and linoleic acid may improve the ability of *M. rileyi* to infect *S. frugiperda* via enhancing the process of conidial germination, appressorium formation, and cuticle penetration.

When the conidia attach to the host cuticle under the appropriate environmental conditions and signals, they germinate and invade the host [49]. Lipids are one of the main components of the body wall, and they may be signaling molecules for conidial germination [22]. Oleic acid and linoleic acid have a generic structure of lipo-chitooligosaccharides (LOCs), and LOCs are perceived at the plasma membrane by receptor-like kinases with extracellular LysM domains [50,51]. For example, LOCs act as regulatory signals of fungal growth and development, and an LOC treatment increased the differentially expressed genes of *A. fumigatus* from 91 to 152 at 30 min and 130 min, respectively [22]. Fungi possess a significant number of LysM-containing proteins [22], suggesting that oleic acid and linoleic acid can be recognized by LysM-containing proteins. Furthermore, fatty acids can be oxidized to produce oxylipins through enzymatic and non-enzymatic reactions [52]. Oxylipin may act as ligands through specific G-protein-coupled receptors (GPCRs), and GPCRs are abundant in filamentous fungi [53,54]. For example, oleic acid promotes GPCR membrane docking and activity as well as related signaling molecules [55]; oleic acid increases the expression of growth- and development-related genes (*Mrap1*, *MrNsdD*, *MrPbs2*, *MrSwi6*, *MrSte12*, and *MrMsn2*) in *M. rileyi* [23]; and oxylipin increases the level of chitin in filamentous fungi [54]. In this study, exogenous oleic acid and linoleic acid up-regulated the expression of growth- and development-related genes, CAT genes, SOD genes, chitin synthase family genes, and cuticle-degrading enzyme-related genes, suggesting that oleic acid and linoleic acid function as signaling molecules for *M. rileyi* conidia. Notably, fatty acid is converted to polyunsaturated fatty acid [56]. Polyunsaturated fatty acids can be metabolized to multiple oxylipins by the cyclooxygenase, lipoxygenase, and/or cytochrome P450 pathways [57]. Oxylipins induce fungal cellular differentiation, including lateral branching, appressorium formation, the yeast-to-hyphal transition, sexual development, and spore release [52,54], suggesting that oleic acid- and linoleic acid-derived oxylipins play a critical role in the regulation of growth and development in *M. rileyi*.

Our and others’ previous work has demonstrated that oleic acid and linoleic acid increase the conidial tolerance to heat, drought, oxidative, osmotic, and cell wall stresses [10,19,24]. These abiotic stresses strongly limit the application of entomopathogenic fungi as biological insecticides [14,58]. Conidial oil suspensions of *M. rileyi*, *M. anisopliae*, and *B. bassiana* can efficiently prevent and control pests in fields [17,59,60]. These results indicate that oleic acid and linoleic acid can be added to oil suspensions of conidia.

## 5. Conclusions

Exogenous oleic acid and linoleic acid improve the stress tolerances and virulence of *M. rileyi*. Their favorable biological characteristics appear to be largely mediated through protecting conidial germination and promoting cuticle infection. These results provide new insights for the development of biopesticides based on *M. rileyi*.

## Data Availability

The original contributions presented in this study are included in the article; further inquiries can be directed to the corresponding authors.

## Supplementary Materials

The following supporting information can be downloaded at: https://www.mdpi.com/article/10.3390/jof10080521/s1, Figure S1: Chemical structures for oleic acid, linoleic acid, stearic acid, eicosane, cetane, palmitic acid, tetracosane, and octacosanol. Figure S2: Growth and development of *M. rileyi*, including conidial germination at 48 h, yeast-like blastospore production at 4 days, mycelial growth at 5 days, and sporulation at 7 days; Table S1: qRT-PCR primers used in this study; Table S2: Colony diameter for all the conidial suspension concentrations; Table S3: Conidial production of *M. rileyi* at different concentrations of oleic acid and linoleic acid; Table S4: The mortality rate of *M. rileyi* against the 3rd, 4th, and 5th instars of *S. frugiperda*; Table S5: The number of *M. rileyi* hyphal bodies in the hemolymph from 72 to 132 h.
