# Supplementary material for: Oleic Acid and Linoleic Acid Enhances the Biocontrol Potential of Metarhizium rileyi
Source: J Fungi (Basel). 2024 Jul 26;10(8):521. doi: 10.3390/jof10080521 (PMC11355944; doi:10.3390/jof10080521)
Supplement: Supplementary file 1 [file jof-10-00521-s001.zip › jof-3093034-supplementary.pdf]

Supplementary information

Supplementary Table S1. qRT-PCR primers used in this study.

Genes	Gene ID	Protein ID	Forward (5'-3')	Reverse (5'-3')
MrChs1	NOR_06275	OAA39437.1	CGATACGAGCTCACAGGGAC	TCGCCTTCGTCATGTATCCG
MrChs2	NOR_02916	OAA46163.1	GTTCTAGATGAGACGCCGGG	GAGTTCCGATGGCTCGTAGG
MrChs3	NOR_03137	OAA46384.1	GAGAAAACGGGCAGCTGTTG	TATGGACGGCGCTAAAGACC
MrChs4	NOR_06155	OAA39735.1	CGTCGATGCATCAACCGAAC	TTGACGCGAGCATTACCCTT
MrChs5	NOR_04139	OAA44411.1	CGATGATCAGACTCGACGCA	GCAGTAGCAGATCCGACCTC
MrChs6	NOR_04125	OAA44397.1	ACGAACGAGGTCTGCATGTT	CAGCGCCAAAACCATGACAA
MrChs7	NOR_04138	OAA44410.1	TATCACCTGCTGGCTCCTCT	CTGCTGCGTAGGCGATTTTC
MrChit1	NOR_07571	OAA36492.1	CGATGGAGTAGACTGGACGC	GCCTTGTAGTGTGTCCGTGA
MrChit2	NOR_07431	OAA36631.1	TGCTCCAACGCAAACATTGG	CGCCTGACAGGACTTGATGT
MrChit3	NOR_07487	OAA36687.1	ATGGGATAAGGAGCTTGCCG	GGGTTGTAGAGCACCCACTC
MrChit4	NOR_05312	OAA41804.1	CGACTCTCTTGCCGCAGTAT	GTCGAGTGCTCCTTCACACA
MrChit5	NOR_03000	OAA46247.1	GATGCATACGCCGCCAATTT	GGCATAGTCGTACGCCATCA
MrPr1	NOR_02420	OAA47930.1	TGAGCGGATTCTCCAGGAGA	GGTCGGCAGATCGTATTCGT
MrPr2	NOR_04608	OAA43241.1	TGGTCAAGTCTGGCGTCTTC	TCGTCACTGTTGGTAGCACC
MrEL	NOR_06300	OAA39462.1	TTTGAGAGCGAGAAGGCCTG	TCCCAATCTCACTCCGCCTA
MrSL	NOR_06146	OAA39726.1	ACCAAGGAAACCAAGGCCAA	GTCCAAGTCCTTGCTGACGA
MrCat1	NOR_06830	OAA38085.1	GAAGTTGACGATGGCGATGC	TCACTTCTCGTCCTGCGCAA
MrCat2	NOR_04290	OAA43715.1	GTTTCCCAGGCCCAGCTATT	CGATTGGCTCTTCGCAATGG
MrCat3	NOR_03956	OAA44228.1	GCATGAACTTCGACGCCATC	GTTGCAAAGCCTGGACACTG
MrSod1	NOR_02650	OAA47014.1	GCGAATGCGTCAGGTGAATC	GGCGCATTCAACCATCCATC
MrSod2	NOR_04943	OAA42094.1	CGGCCTTTCTCTTCCTGGTT	CTCACTTGGACGGCCAGATT
MrSod3	NOR_04776	OAA42645.1	CCCACAGGGCATAAAGGTGT	GCTTGATATCGTCCTGCCGA
MrSod4	NOR_05685	OAA40597.1	GCTACATACGACTCCAGCCC	TCAACGATCCGTCCTCGCAA
MrPbs2	NOR_02444	OAA46808.1	GAGCCTGCTGTTGGAACTCT	ACATCTTCGGGTGCGGAAAT
MrMsn2	NOR_04557	OAA43190.1	ACACCCCATCTGCTCCATTG	CAGGTGTTCTTGACGACGGA
MrSwi6	NOR_02859	OAA46106.1	ACGCCACCCAGATTCTCAAG	TTTTCGTGCTCGCCTGTTTG
MrNsdD	NOR_03486	OAA45697.1	ATTGGGTCAAACTCGCGGAT	TGACCGCTTGATTGTGTCGA
MrSte12	NOR_06783	OAA38038.1	TCGGCTCCGGCATATCTAGA	GATACGTCTCGGTGCTCTGG
MrTEF	NOR_02491	OAA46855.1	GTCATCGTCCTCAACCATC	CAGTCTCAACAGCCTTACC
MrTUB	NOR_00286	OAA51693.1	GGCAAGGTCGCTATGAAG	CTGGATGGAGGTAGAGTTAC

Note: Chs, Chitin synthase; Chit, Chitinase; Pr, Protease; EL, Extracellular lipase; SL, Secreted lipase; Cat, Catalase; Sod, Superoxide dismutase; Pbs2, mitogen-activated protein kinase kinase Pbs2; Msn2, zinc finger DNA-binding protein Msn2; Swi6, cell cycle box binding transcription factor MrSwi6; NsdD, GATA-type transcription factor NsdD; Ste12, transcription factors Ste12; TEF, transcription elongation factor; TUB, β-tubulin.

Supplementary Table S2. Colony diameter for all the conidial suspension concentrations.

Concentration	Time	Water	OA	*P* value	LA	*P* value
10^5conidia/mL	7 d	6.7 ± 0.4	7.8 ± 0.1**	t-test, $P = 0.0004$	9.2 ± 0.5$^{\#\#}$	t-test, $P = 0.0004$
	9 d	7.9 ± 0.2	9.7 ± 0.2**	t-test, $P = 0.0018$	10.7 ± 0.5$^{\#\#}$	t-test, $P = 0.0007$
	11 d	9.7 ± 0.0	11.4 ± 0.4*	wilcox test, $P = 0.0232$	11.9 ± 0.4$^{\#}$	wilcox test, $P = 0.0232$
10^6 conidia/mL	5 d	6.8 ± 0.1	7.6 ± 0.3*	t-test, $P = 0.0211$	8.4 ± 0.7$^{\#\#}$	t-test, $P = 0.0065$
	7 d	7.3 ± 0.1	9.0 ± 0.2**	t-test, $P = 0.0002$	9.9 ± 0.5$^{\#\#}$	t-test, $P = 0.0018$
	9 d	9.0 ± 0.1	11.0 ± 0.4**	t-test, $P = 0.0013$	11.8 ± 0.7$^{\#}$	wilcox test, $P = 0.0248$
	11 d	10.9 ± 0.4	13.7 ± 0.5**	t-test, $P = 0.0016$	13.5 ± 0.6$^{\#\#}$	t-test, $P = 0.0021$
10^7 conidia/mL	5 d	7.1 ± 0.1	8.4 ± 0.2**	t-test, $P = 0.0008$	9.7 ± 1.0$^{\#\#}$	t-test, $P = 0.0020$
	7 d	8.8 ± 0.1	10.8 ± 0.9*	wilcox test, $P = 0.0248$	11.9 ± 1.0$^{\#}$	wilcox test, $P = 0.0.0248$
	9 d	10.4 ± 0.2	11.7 ± 0.2*	t-test, $P = 0.0189$	13.7 ± 1.2$^{\#}$	t-test, $P = 0.0107$

Note: LA, linoleic acid; OA, oleic acid. $^{*}P < 0.05$ and $^{**}P < 0.01$ when OA compared to the Water treatment. $^{\#}P < 0.05$ and $^{\#\#}P < 0.01$ when LA compared to the Water treatment.

Supplementary Table S3. Conidial production of *M. rileyi* at different concentrations of oleic acid and linoleic acid.

Concentration	10^6 conidia/mL		10^7 conidia/mL	
	OA	LA	OA	LA
0.00	10.6 ± 0.4	10.6 ± 0.4	14.3 ± 0.9^{b}	13.9 ± 1.2^{b}
0.01%	10.7 ± 0.6	10.4 ± 0.8	14.3 ± 1.7^{b}	16.2 ± 1.7^{ab}
0.05%	11.4 ± 0.2	11.0 ± 0.3	14.0 ± 0.6^{b}	17.1 ± 1.5^{ab}
0.10%	13.2 ± 0.4	11.8 ± 0.5	14.7 ± 1.0^{ab}	20.1 ± 2.1^{a}
0.20%	12.3 ± 0.4	12.1 ± 0.8	19.3 ± 1.4^{a}	17.8 ± 1.8^{ab}
0.40%	10.6 ± 1.0	11.1 ± 0.9	12.0 ± 2.2^{b}	14.0 ± 1.2^{b}
P value	0.247	0.052	0.048	0.012

Note: LA, linoleic acid; OA, oleic acid. Different letters indicate statistical significance ($P < 0.05$).

Supplementary Table S4. The mortality rate of *M. rileyi* against the 3rd, 4th, and 5th instars of *S. frugiperda.*

Treatments	3rd	4th	5th
0.02% Tween 80	0.0 ± 0.0%^{d}	0.0 ± 0.0%^{c}	0.0 ± 0.0%^{c}
OA	65.7 ± 1.3%^{c}	0.0 ± 0.0%^{c}	0.0 ± 0.0%^{c}
LA	64.8 ± 1.3%^{c}	0.0 ± 0.0%^{c}	0.0 ± 0.0%^{c}
Mr	86.4 ± 3.7%^{b}	40.0 ± 1.8%^{b}	31.3 ± 2.9%^{b}
Mr+OA	99.1 ± 1.3%^{a}	54.4 ± 2.3%^{a}	48.5 ± 4.3%^{a}
Mr+LA	99.1 ± 1.3%^{a}	52.2 ± 3.4%^{a}	48.5 ± 4.3%^{a}
P value	0.0067	0.0058	0.0057

Note: LA, linoleic acid; OA, oleic acid; Mr, *M. rileyi.* Different letters indicate statistical significance ($P < 0.05$).

Supplementary Table S5. The number of *M. rileyi* hyphal bodies in the hemolymph from 72 to 132 hours.

Time	CK	OA	*T* value; *P* value	LA	*T* value; *P* value
72 h	0.0 ± 0.0	0.0 ± 0.0	-	0.0 ± 0.0	-
84 h	0.0 ± 0.0	3.2 ± 2.6*	wilcox test, $P = 0.0026$	0.0 ± 0.0	-
96 h	0.0 ± 0.0	8.2 ± 3.0**	wilcox test, $P = 0.0027$	1.6 ± 0.8$^{\#\#}$	wilcox test, $P = 0.0024$
108 h	2.6 ± 1.4	15.0 ± 6.8**	wilcox test, $P = 0.0044$	7.0 ± 1.7$^{\#\#}$	t-test, $P = 0.0035$
120 h	8.6 ± 3.9	23.4 ± 8.8*	t-test, $P = 0.0153$	16.4 ± 6.7	t-test, $P = 0.0781$
132 h	15.4 ± 8.6	64.8 ± 14.4**	t-test, $P = 0.0004$	47.8 ± 14.6$^{\#\#}$	t-test, $P = 0.0051$

Note: LA, linoleic acid; OA, oleic acid; $^{*}P < 0.05$ and $^{**}P < 0.01$ when OA compared to the CK treatment. $^{\#\#}P < 0.01$ when LA compared to the CK treatment.

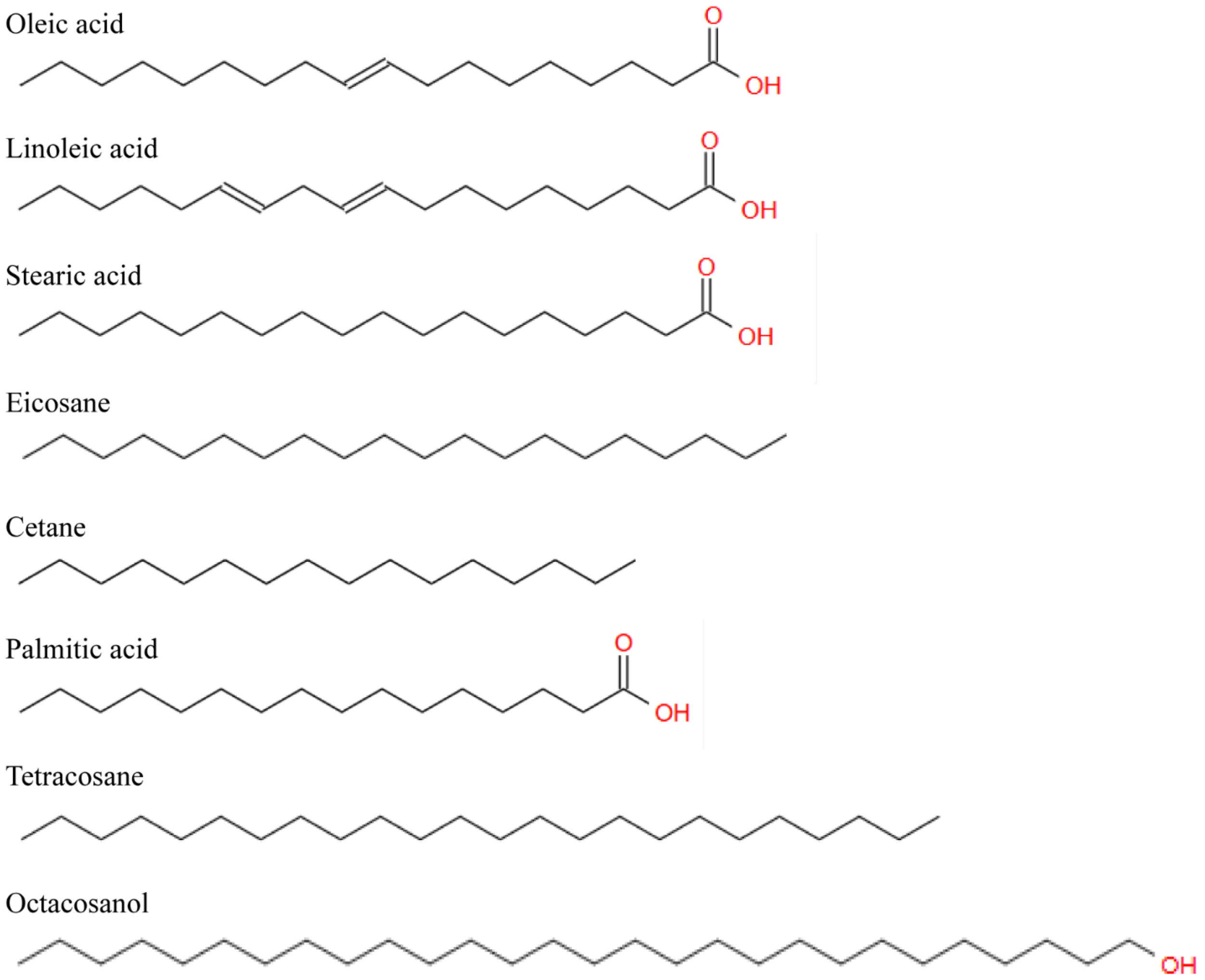

Supplementary Figure S1. Chemical structures for oleic acid, linoleic acid, stearic acid, eicosane, cetane, palmitic acid, tetracosane, and octacosanol.

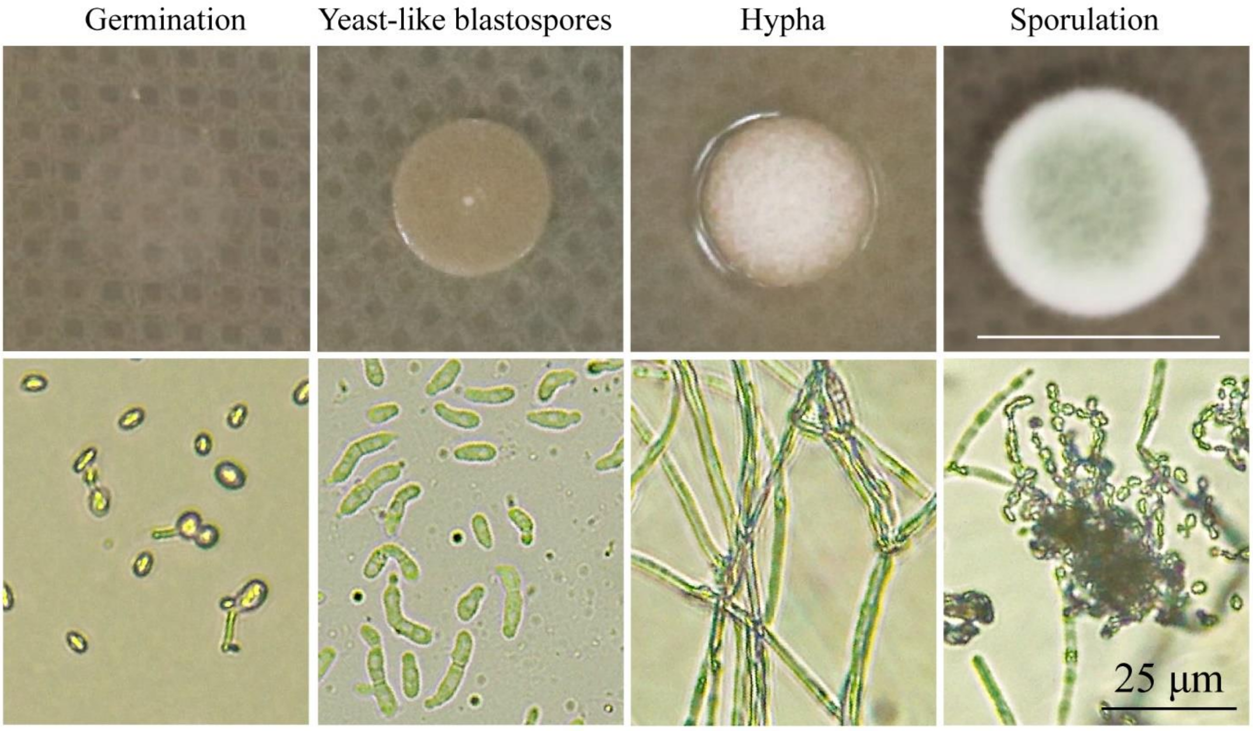

Supplementary Figure S2. Growth and development of *M. rileyi*, including conidial germination at 48 h, yeast-like blastospore production at 4 days, mycelial growth at 5 days, and sporulation at 7 days. The media plates were incubated at 25℃ with a photoperiod consisting of 12L:12D cycle in a culture chamber.
